# Unlocking the secrets of the microbiome: exploring the dynamic microbial interplay with humans through metabolomics and their manipulation for synthetic biology applications

**DOI:** 10.1042/BCJ20210534

**Published:** 2023-06-28

**Authors:** Howbeer Muhamadali, Catherine L. Winder, Warwick B. Dunn, Royston Goodacre

**Affiliations:** Centre for Metabolomics Research, Department of Biochemistry, Cell and Systems Biology, Institute of Systems, Molecular and Integrative Biology, University of Liverpool, Liverpool L69 7ZB, U.K.

**Keywords:** metabolites, metabolomics, microbiome, multi-omics, synthetic biology

## Abstract

Metabolomics is a powerful research discovery tool with the potential to measure hundreds to low thousands of metabolites. In this review, we discuss the application of GC–MS and LC–MS in discovery-based metabolomics research, we define metabolomics workflows and we highlight considerations that need to be addressed in order to generate robust and reproducible data. We stress that metabolomics is now routinely applied across the biological sciences to study microbiomes from relatively simple microbial systems to their complex interactions within consortia in the host and the environment and highlight this in a range of biological species and mammalian systems including humans. However, challenges do still exist that need to be overcome to maximise the potential for metabolomics to help us understanding biological systems. To demonstrate the potential of the approach we discuss the application of metabolomics in two broad research areas: (1) synthetic biology to increase the production of high-value fine chemicals and reduction in secondary by-products and (2) gut microbial interaction with the human host. While burgeoning in importance, the latter is still in its infancy and will benefit from the development of tools to detangle host–gut-microbial interactions and their impact on human health and diseases.

## Introduction

Metabolites have many important roles in the reproduction, growth and survival of all biological organisms. These small molecules are the building blocks for other biochemicals including proteins, RNA and DNA. Through their metabolic interconversion they provide the components for many biological processes including energy production and the synthesis of lipids used for cell membrane construction. Metabolites act to regulate biochemical processes through protein regulation including post-translational modifications (for example, see [1]) and allosterism [2], epigenetics [3] and signalling (e.g. sphingosine-1-phosphate [4]).

The qualitative and quantitative composition of metabolites in a single sample type (biofluid, cell, tissue) or whole organism is known as the metabolome and contain hundreds or low thousands of detected water-soluble and lipid metabolites (more accurately referred to as lipidomics [5]) of diverse chemical structures and physical properties. The concentration of metabolites in many of these metabolomes are highly dynamic with changes following an environmental perturbation observed in seconds to minutes; for example, strenuous exercise will lead to the generation of increased lactate levels via anaerobic respiration in muscle and subsequently blood within seconds/minutes. The dynamics of metabolism (and other metabolite-containing biochemical processes) allows the dynamic phenotype to be measured at the metabolite level, sometimes called the metabolic phenotype, which is a measure of the genotype × environment interaction. The study of the metabolome at the global level with the reporting of data for hundreds to low thousands of metabolites is called metabolomics and is analogous to other omic disciplines including proteomics and transcriptomics [6–8].

### Metabolomics

Metabolomics is applied as a research discovery tool with the objective of untargeted [8] approaches being to detect, and report normalised relative peak areas for as many metabolites as can be reliably measured, though in some applications the concentration of the metabolite can be reported for tens or low hundreds of metabolites [9]. Univariate and multivariate analysis of the acquired data is subsequently applied to define the biologically important metabolites and generate a hypothesis for further biological testing. The number of metabolites for which data are reported is dependent on the metabolome of the sample type, the sample extraction method employed as well as the analytical platform applied. Of the most frequently applied analytical platforms nuclear magnetic resonance (NMR) spectroscopy typically reports on tens of metabolites, gas chromatography–mass spectrometry (GC–MS) reports on low hundreds of metabolites and liquid chromatography–mass spectrometry (LC–MS) reports on high hundreds to low thousands of metabolites. Importantly, the chemical structure of metabolites to be detected are not known or are known but are not specifically targeted in these discovery studies, this is deliberate to ensure there is no biological bias in the detection of a high number of metabolites [10]. Instead, data are collected to allow the structural identification of metabolites after data acquisition; this provides limitations as will be discussed later. Semi-targeted (hybrid) studies [11] are also a discovery tool but a target list of metabolites that are expected to be measured are predefined. The applied assay(s) allows collection of data to confirm the presence of each metabolite on the target list (cf. untargeted assays where data are collected to identify the metabolite structure). This reduces the number of metabolites reported but allows for more confident metabolite identifications and a quicker process to biological interpretation; this approach reports normalised peak areas and not metabolite concentrations though some hybrid approaches do report metabolite concentrations for hundreds of lipids (as an example see [12]). In comparison, targeted analytical assays focus on a few metabolites known to be of biological relevance (possible revealed in a discovery metabolomics study) and provides high confidence of metabolite identification and reported concentrations. The analytical assay should be validated to demonstrate the accuracy and precision of data, sample stability and calibration parameters (limit of detection, linear calibration range) to meet defined acceptance criteria (the latest recommendation by the Food and Drug Administration are available at https://www.fda.gov/media/162903/download).

**Figure 1.**
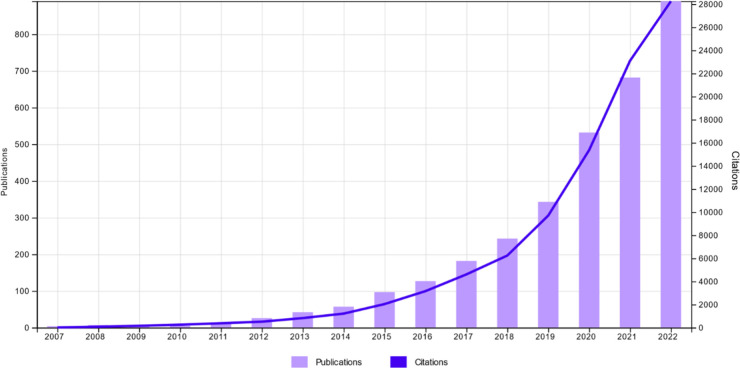
Growth in studies that combine metabolomics and microbiome work since 2007. Number of publications (purple bars) versus number of citations (blue line) of these publications on the application of metabolomics technologies in the area of microbiome from 2007 to 2022 (from Thomas Reuters’ ISI Web of Science Core Collection, using the keywords [metabolomic* and microbiome*]).

Metabolomics is applied across the biological sciences from the study of microbial systems [13] and their interaction with other biological systems (e.g. human gut microbiome [14]), the study of the environment and its biological species (including plants [15] and aquatic systems [16]) to the study of mammals with a perhaps unsurprising large focus on humans [17]. Metabolomics approaches are normally applied to answer one of two different types of question; either to identify prognostic or diagnostic biomarkers of health or disease or to identify new biochemical processes/pathophysiological mechanisms. The number of applications are growing rapidly as demonstrated by the number of papers published each year (Figure 1 demonstrates the number of metabolomics microbiome publications during the last 20 years) as well as the number of studies being submitted to metabolomics data repositories such as Metabolights [18] which provides specific statistics on data growth over time (https://www.ebi.ac.uk/metabolights/statistics).

### The metabolomics workflow

**Figure 2.**
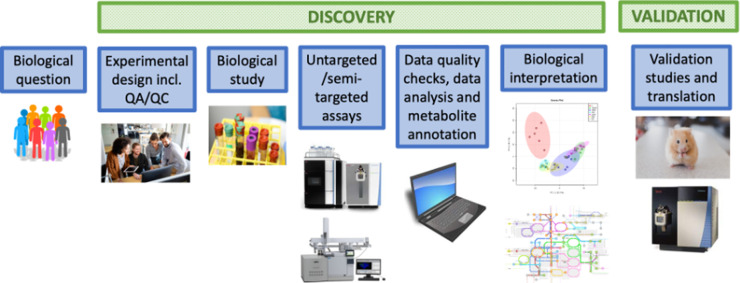
The metabolomics study workflow. Once the biological question is clearly defined the study can be designed to plan all aspects of the workflow robustly. The biological study can be performed with biological sample collection and storage. Biological samples are extracted and analysed applying untargeted/semi-targeted assays. Raw data are processed to construct a data matrix, the data are quality checked, metabolites are annotated, data are statistically analysed and the results are biologically interpreted. A hypothesis is created and tested in the validation study with further work to develop through to translation. In some cases, the metabolites of interest are known (see synthetic biology discussion) and the discovery part of the workflow is not required.

The generalised metabolomics workflow is described in Figure 2. All parts of this workflow are important and the formation of this pipeline should be designed appropriately at the start of the study to ensure that the biological question of interest can be answered in a robust way. One important aspect is the choice of analytical platform to apply for data collection in untargeted studies. Each analytical platform has advantages and limitations related to sensitivity and quantitation capabilities, number of detected metabolites and ease/difficulty of metabolite annotation/identification. The three most commonly applied platforms are LC–MS, GC–MS and NMR spectroscopy. In this review, we will focus on the use of GC–MS and LC–MS, though it should be noted that many other analytical platforms can be applied including direct infusion mass spectrometry (DIMS) [19,20], as well as infrared spectroscopy [21] and Raman spectroscopy [22] which can generate chemical profiles and images of biological systems. There are a range of experimental processes to be carefully considered when applying LC–MS and GC–MS platforms in discovery-based metabolomics research and these will be discussed below.

### Considerations when applying LC–MS and GC–MS in discovery metabolomics studies

LC–MS and GC–MS are commonly applied in discovery-based metabolomics research because they allow for the detection of hundreds or low thousands of metabolites across many different areas of metabolism at concentrations from sub-micromolar to millimolar. This meets the requirements of discovery metabolomics which has the goal to detect and report as many metabolites as is possible. However, there are many considerations when applying these platforms.

#### Sample preparation

The goal of discovery metabolomics is to report high quality data for as many metabolites as is feasible; the aim of the sample preparation procedure is to prepare a sample extract containing as many metabolites as possible. For liquid samples the process (i) removes larger biochemicals (e.g. proteins) or other metabolites classes (e.g. lipids in a water-soluble metabolite assay) which can interfere with the assay (particularly the chromatography) or cause instrument problems and (ii) transfers metabolites into an instrument-compatible solvent. For cells and tissues, a similar process is performed though additional steps of tissue homogenisation and cell lysis are required to facilitate the release of metabolites. It is essential to consider the metabolic activity of the sample type; samples containing enzymes are defined as metabolically active and, therefore, metabolism is rapidly inhibited (quenched) [23,24] at the time of sample collection so to provide a sample representative of the phenotype and environment to be studied. In general, biofluids and cell media are normally not considered metabolically active, while cells and tissues are metabolically active. However, biofluids can be metabolically active if they contain cells. Monophasic extraction processes use one or multiple miscible solvents and these solvent compositions are optimised to allow a wide range of metabolites to be extracted [25]. However, in some cases the application of biphasic extractions is applied to simplify the sample extract to be analysed, the best example is use of a biphasic extraction to separate lipids in to one phase and water-soluble metabolites in to a second phase with each phase analysed applying a different analytical assay [26]; this approach allows chemical simplification of the sample extract for assays while allowing detection of the largest number of metabolites when multiple assays are applied. When applying a more targeted analytical assay, a few biologically important metabolites are studied, further sample preparation can be performed to further isolate the target metabolites and remove other metabolites and lipids not of biological interest (e.g. use of solid phase extraction to remove lipid molecules [27]).

#### Sample throughput and sample size

To provide statistical robustness multiple biological replicates for each biological class are studied. As a general rule, the greater the biological variability between replicates then the higher the number of biological replicates are required to provide statistical significance. For example, the growth of yeast in a laboratory with the same defined culture media, where there is one genotype and with very reproducible growth conditions then a minimum of six biological replicates is usually recommended. However, if studying humans where there is a different genotype for each sample and different environmental conditions (e.g. diet, exercise and exposures) for each subject then a much larger number of biological replicates is required; typically hundreds or thousands. Metabolomics has developed to provide medium-to-high throughput assays, typically of less than 20 min in length which allows more than 70 samples to be analysed each day and the analysis of hundreds of samples each week. Short assay lengths do lead to significant co-elution of metabolites, whereas the application of longer assay times can lead to better separation and less co-elution but at the expense of throughput [28]. Some assays technically have to be longer in length, comprehensive GCxGC–MS assays are typically 30–40 min in length [29].

#### Instrument stability/data drift

Drift in measured data including response, mass accuracy and retention time (RT)/retention index (RI) is important to consider in medium to high throughput assays where data for hundreds or thousands of samples are acquired. Drift in these types of data can lead to the same metabolite being reported multiple times, incorrect structural annotations and a reduced statistical power because of the increased variation in the data. In NMR spectroscopy, data collected over days/weeks are very reproducible in terms of response and chemical shift because samples are placed in a glass tube which does not physically interact with the instrument. In GC–MS and LC–MS, the extracted sample components physically interact with the instrument (most notably the stationary phase used for chromatography) and can lead to the accumulation of dirt and result in changes/poorer stability of data. The most common form of drift is observed for response with RT and mass accuracy being relatively stable across time with appropriate instrument maintenance and calibration [30]. Dirtying of the system means that voltages can change and so ion transmission can be reduced. The undesirable effect of this is a change in the response over time. To minimise this drift, regular maintenance of the instrument is required. The frequency of cleaning is dependent on sample throughput, sample complexity and instrument and injection volume [30]. It is important to define the frequency of cleaning, this can be evaluated via the application of different types of QC samples to monitor drift/stability within and between studies as defined below.

#### Quality assurance and quality control processes

The ability to report the quality of metabolomics data and to remove poor quality data before data analysis is an important process of any study collecting omics data. Quality assurance (QA) processes are those applied prior to data collection and include training, standard operating procedures (SOPs) as well as instrument maintenance/tuning/calibration. Quality control (QC) processes are those applied during and after data collection to evaluate the quality of the data and to remove poor quality data from a dataset. In metabolomics, QC processes revolve around the use of QC samples of which there are many types including process and extraction blank samples, pooled QC samples, standard reference materials and internal standards [31,32]. Through these data one can provide evidence of the quality of data collected for each metabolite, remove data for metabolites which do not meet a certain predetermined quality criteria, remove data where a significant contribution is observed in the blank sample and is, therefore, not of biological origin and remove outlier samples [31]. Through the application of these processes one can also remove poor quality data which may impact on subsequent data analysis and demonstrate the quality of the data prior to the application of data analysis and most importantly biological interpretation. Appropriate reporting standards have recently been developed for community use [33].

#### Metabolite annotation/identification

Discovery phase untargeted metabolomics operates by analysing samples without knowing which metabolites will be detected; i.e. a target list is not defined and data collected to confirm the chemical structure of the metabolite target (as occurs for targeted chemical assays) is not performed. Instead, data are collected to allow peak areas to be reported and metabolites to be annotated/identified after data acquisition. In GC–MS where electron ionisation (EI) is applied, gas-phase fragmentation of all metabolites is automatically performed as a consequence of the ionisation process. The fragmentation mass spectrum is characteristic of the metabolite's chemical structure (weaker covalent bonds tend to fragment first) and so can be used for identification, which is required to provide a biological interpretation. Fortunately, this EI fragmentation process is consistent across all instruments and laboratories worldwide, and mass spectral libraries containing many thousands of metabolites are available. The RT can also be normalised across laboratories through the addition of retention indices. The result is that a larger proportion of metabolites that can be identified relatively easily applying GC–MS when compared with LC–MS. In LC–MS, the electrospray ionisation (ESI) process can generate many different metabolite features for the same metabolite with different *m/z* and the same RT and so the detection of one thousand metabolites can result in the reporting of >10 000 metabolite features [34,35]. To aid the identification of metabolites in LC–MS additional MS/MS data can be collected, and in most cases are mandatory for metabolite identification. Gas-phase fragmentation can be collected for all metabolites using data independent analysis (DIA) though the complexity of mass spectrometry data and co-elution of multiple metabolites can make the computational deconvolution of mass spectra difficult and inaccurate. Traditionally, data dependent analysis (DDA) is more commonly applied; however, it is difficult to collect MS/MS data for all metabolites which are informative and lead to metabolite identification with this approach. Potential reasons for this include, the use of different collision energies (cf. GC–MS where there is one collision energy (typically in EI this is 70 eV)), the lower coverage of metabolites in MS/MS libraries and the fact that most libraries report [M + H]^+^ and [M − H]^−^ adduct mass spectra, whereas the experimentally collected MS/MS mass spectra may be (and commonly is) for another adduct or in-source fragment and so no match is possible. With this example the use of *in silico* approaches to use MS/MS mass spectral similarity between mass spectra for knowns and unknowns is applied (for example, GNPS [36]). One final parameter to consider with respect to LC–MS and the annotation/identification pipeline is RT. RT is instrument, column, mobile phase and gradient elution dependent, and as there are many different columns commercially available (even for the same phase) and different gradient elution applied, RTs are not standardised across different assays/laboratories and so RTs are not readily comparable/transferable across laboratories. The result of this is that although the RT is measured for all detected features the use of RT in the annotation/identification process is limited to in-house libraries, although the use of RT mapping [37,38] and *in silico* prediction [39,40] has been applied, and over time will be improved. As the confidence of annotations can vary considerably, the methods and evidence should be provided when reporting along with reporting standards nomenclature (e.g. the metabolomics standards initiative; MSI [41]). High confidence reporting of chemical structures is defined as identification and, as an example for LC–MS, applies MS, MS/MS and RT matching of experimental data to data collected for chemical standards (MSI Level 1). Lower confidence reporting is defined as annotation and typically only matches one or two parameters to a chemical standard held in a third party library constructed with chemical standards or to theoretical metabolite data in databases, typically using MS1 only data (MSI Levels 2 and 3).

Above we discussed why, how and considerations of applying metabolomics in biological research. While many reviews focus on the clinical applications of metabolomics and others on plant metabolomics, we recognise that microbial metabolomics is an important and broad discipline. Microbes (bacteria, yeast and fungi) are becoming increasingly important as microbial cell factories to produce fine chemicals for so called ‘white biotechnology’ where sustainable production of metabolites and proteins is important, thus we first focus on this area of synthetic biology. The second area that is captivating is how microbial communities interact with humans and thus we shall also focus on the application of metabolomics for investigating human microbiome interactions and their role in health.

## The application of metabolomics in synthetic biology

There are many areas where metabolomics is used within synthetic biology and this section briefly covers the main three strategies. These include targeted analysis of specific metabolites, metabolomics in the traditional untargeted manner, as well as considerations of dynamics in biological systems by adopting methods that measure, for example, carbon flux through pathways.

**Figure 3.**
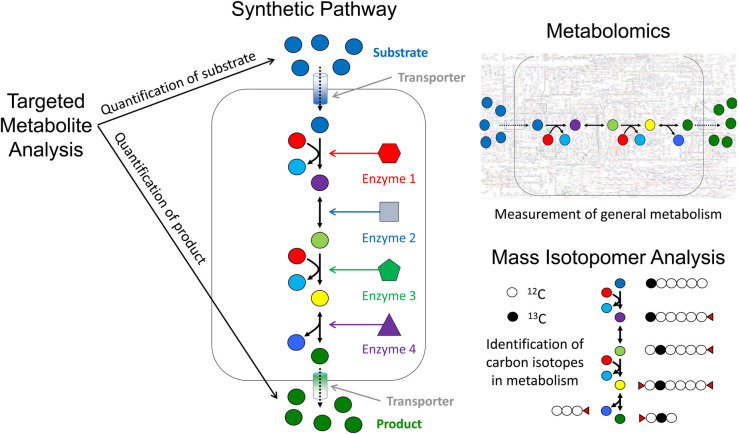
An illustration of the different ways in which a synthetic pathway can be analysed using metabolomics and related methods. This can be performed using *Targeted Metabolite Analysis* of substrates and products, employing *Metabolomics* where general metabolism of the host and pathway are measured (both inside and outside the cell), or via *Mass Isotopomer Analysis* to follow the flow of specific isotope labelled molecules — here with the example being the replacement of ^12^C with ^13^C.

Central to systems biology and hence synthetic biology is having a metabolic network model that describes the pathways in the organism and includes the new synthetic pathways or those existing pathways that need to be enhanced. These are often achieved by genome-scale metabolic reconstructions where the whole genome is translated into metabolic networks by bibliographic searches, knowledge in other pathways from, for example, BioCyc (https://biocyc.org) or KEGG (https://www.kegg.jp), where one can gain species specific information on genes that encode enzymes. This leads to knowledge of reactions and the metabolites involved (substrate(s) and product(s)) and thus pathways can be formalised, linked and thereby integrated into metabolic network reconstructions; finally, these are often agreed upon by jamborees [42,43]. Often there are gaps within reconstructions and bioinformatic tools which can be useful for metabolite annotation [44]. We shall see for all types of metabolomics investigations within synthetic biology metabolic reconstructions are an essential part of the analytical pipeline (Figure 3).

### Targeted analysis for synthetic biology

Over the last decade or so the optimisation of new synthetic biology pathways has embraced automated Design–Build–Test–Learn (DBTL) pipelines as this marriage of computational and laboratory automation has enhanced production through rapid pathway optimisation.

Let us consider the construction of a new pathway where genes encoding a series of enzymes are combined to produce a (bio)chemical that is not normally produced by the host or is produced but at very low levels. Within the DBTL cycle the *Design phase* is used to construct the pathway *in silico* and genes that encode specific enzymes are selected. These genes can be from different organisms such that the pathway is ‘chimeric’ at least from the genetic point of view, but the pathway undergoes a series of chemical reactions that are enzymatically mediated. Within the automation process computational design of experiments, such as Latin square, are used to generate a series of pathways that cover different vector backbones, promoters, copy numbers of genes, gene positions and intergenic regions. The assembly instructions are then sent to the *Build phase*, often within a robotic system, that first synthesises the DNA, assembles the pathway and then sequences the whole cassette to verify the DNA sequence is correct. Once the pathway is constructed it is inserted into the host it was designed for. Note that the design phase ensures any introns are removed if the original gene is eukaryotic and the host bacterial. In addition, the codon usage is adjusted appropriately based on the GC:AT ratio of the host that will be synthesising the enzymes for this new pathway. The *Test phase* is where accurate and precise metabolite quantification of the substrate(s) and product(s) is needed and targeted metabolite analysis with absolute quantification is used. After this the last part of the cycle is the *Learn phase* where machine learning is used to associate the pathways that were constructed with the level of the product. In this phase ‘rules’ are generated that informs one about the best gene position, promoter and copy number as well as intergenic regions. These rules are fed to the next design phase such that new pathways are engineered, constructed and tested. The cycle continues until the desired level/yield of the substrate is generated.

An example of this automated and iterative DBTL pipeline is illustrated by Carbonell et al. [45] who aimed to synthesise (2*S*)-pinocembrin in *Escherichia coli*. This flavonoid was generated by a pathway that started at l-phenylalanine and contained four enzymes: three were from *Arabidopsis thaliana* and one from *Streptomyces coelicolor*, highlighting the need to remove introns from the plant and adjust the codon bias as *S. coelicolor* is GC rich in comparison with the AT rich *E. coli*. Of the possible 2592 pathways that could be constructed the design phase selected 16 pathways, all of which could be constructed/built and subsequently tested. All 16 pathways were functional, and the level of (2*S*)-pinocembrin varied from 2 to 140 µg/l. The learn phase discovered four rules that were used to generate a further series of pathways and the best pathway generated 6.6 mg/l of the flavonoid, which was a 46-fold increase in pinocembrin production. Following this, process optimisation was performed which involved additional screening for optimal chassis, growth media and *E. coli* strain resulting in a final titre of up to 88 mg/l. This level is industrially competitive and highlights that this metabolic engineering DBTL process is an efficient way to optimise pathways in a very rapid manner such that they are suitable for automated biomanufacturing.

In the above example, targeted metabolite analysis was used which employed LC–MS using a triple quadrupole mass analyser for measuring selected multiple reaction monitoring (MRM) transitions so that accurate absolute quantification of pinocembrin was achieved in an automated manner. Another nice example in *E. coli* is that in just 2 DBTL cycles a 21% increase in 1-dodecanol levels was achieved which is six-fold greater than previously reported [46]. Finally, the adoption of DBTL for synthetic biology is not limited to bacterial, yeast or eukaryotic cellular systems but the same principles can also be applied to plants as recently highlighted by Pouvreau et al. [47] who described the convergence of plant metabolic engineering and plant synthetic biology and the need to adopt the DBTL cycle concept.

### Metabolomics for synthetic biology

When targeted metabolite analysis is used it predominantly focusses on the product, although the substrate concentration and any intermediates can also be measured. However, this focus does not consider general metabolism or the flow of carbon from the substrate to the product. Metabolomics can be very powerful to allow pathways to be debugged so that optimal carbon flow to the product is achieved or can be used to identify nutrient limitations in culture media. Indeed, metabolomics has been an essential element within synthetic biology of secondary metabolism for the generation of natural products [48,49].

Many products of synthetic biology are not small molecules and can be macromolecules like proteins or RNA. Within the biopharmaceutical area the efficient production of recombinant proteins in host cells is important for targeted immunotherapy. Many systems currently use Chinese hamster ovary (CHO) cells as the host as this allows correct glycosylation of the product. In a study by Sellick et al. [50], CHO cells were engineered to produce the product IgG4. Metabolomics of both the intracellular CHO metabolites and extracellular metabolites were measured using GC–MS over an 11-day production run. GC–MS was selected as the analytical approach because it measures predominantly central carbon and nitrogen metabolism (viz. sugars, sugar phosphates, amino acids, organic acids and fatty acids). Overlaying the concentrations of these metabolites onto metabolic maps of CHO metabolism allowed the identification of substrates that depleted either quickly or slowly, as well as highlighting metabolites that accumulated in the culture medium. The knowledge from this metabolomics-based strategy allowed the design of novel low-cost nutrient feeds. When tested these resulted in an increase in cell biomass by 35% and the antibody titre doubled.

### Dynamics in systems biology

Within synthetic biology, targeted metabolite analysis and metabolomics are valuable approaches as they provide information on changes during the DBTL cycles that can be linked to the productivity of the metabolite or protein end-product. However, while samples can be taken at various time points to follow changes in the levels of metabolites during the fine chemical or biopharmaceutical production process that can be linked to metabolic pathways, they fail to tell you about the *flow of metabolites*. In the traditional sense metabolomics takes snapshots of events frozen in time and, therefore, any dynamics within the process are lost. This is especially the case as the output of metabolomics experiments are the concentration of metabolites and not the rate at which that metabolite is produced or consumed. In addition, in many circumstances the level of metabolites might be the same (or very similar) between different conditions, or very small in quantity yet important, because the flow of carbon through some pathways is very fast. This is to be expected as metabolic fluxes (at least as exemplified by glycolysis in trypanosomes) are not regulated by gene expression alone and are allosterically regulated and thus the rate that glucose is turned into pyruvate is <1 s [51]. All of these points provide further rationale for pursuing fluxomics as well as metabolomics, as these approaches measure rates of metabolic reactions in biological systems. This is important as this helps differentiate hierarchical regulation of enzyme synthesis or activity which is governed by activators or repressors at the transcriptional or translational levels, from allosteric regulation of enzyme activity which is governed by effector molecules (usually small molecules (i.e. metabolites) that non-covalently bind to enzymes altering activity). The speed of this regulation is extremely rapid, dependent on the organism — prokaryotic *versus* eukaryotic — and is nicely captured in this snapshot by Shamir et al. [52].

Using a motorway analogy of metabolism and the role of metabolites, as described by Jang et al. [53] and as applied by these authors in [54], let us consider a motorway (autobahn or highway) where cars, vans and lorries, as well as the different makes of vehicle, represent different metabolites. If we were to stand on a bridge/flyover, we would observe the flow of vehicles as the vehicles on the motorway move. Taking a photo rather than a video belies the true event that is ongoing. Now let us consider complex motorway junctions such as the Spaghetti Junction in Birmingham in the U.K. In such junctions (or hubs), we see many intersections of roads and this we can think of as a metabolic network. If we could measure the speed and/or destination of the vehicles (metabolites) through such junctions/hubs we would be able to enhance our understanding of metabolism.

The ability to measure speed of processes through metabolic pathways allows one to understand where, for example, carbon is being distributed. For systems biology if we were to measure where carbon goes and it were to go towards the wrong endpoint, one can debug metabolic pathways by employing rationale metabolic engineering of the system to redirect the carbon flow to the correct place; for example, towards a precursor for biotechnological and pharmaceutical industries. This of course necessitates that metabolic networks can be modelled computationally and combined with experimental data, and this is essential for understanding the flow of carbon through metabolic networks.

Stable atomic isotopes in key metabolites can be used to follow/track the flow of carbon, nitrogen and indeed other stable isotopes, in metabolic pathways. For example, if changes in central carbon metabolism are thought to occur and the system uses glucose as the main carbon supply then dynamics within the system can be probed using glucose labelled with ^13^C isotopes. Carbon is found in nature predominantly as ^12^C (98.9%; which possesses six protons and six neutrons) with a small amount of the stable isotope ^13^C (1.1%; which by contrast possesses six protons and seven neutrons), and an even smaller amount of radioactive ^14^C (six protons and eight neutrons). Mass isotopomers are molecules where each molecule with *n* carbon atoms has *n *+ 1 carbon mass isotopomers. Molecules labelled with ^13^C can be differentiated from their natural equivalent by mass differences measured via MS, or via chemical shift variations in NMR spectrometry [55].

As detailed by us in [56] for general labelling uniform labelled glucose ([U-^13^C_6_]-d-glucose) can be used as a starting substrate where all six carbons are ^13^C (rather than ^12^C). We refer to these two different types of glucose as mass isotopomers (as defined above) as while they have the same empirical formula and structure, for ([U-^13^C_6_]-d-glucose) this molecule has a mass of 186 compared with 180 in the natural (^12^C) isotope of glucose. For studying glycolysis and TCA, labelling the first carbon ([1-^13^C_1_]-d-glucose) is useful and depending on carbon flow pyruvate will become labelled with ^13^C and thus have a mass difference of 1. The fate of pyruvate — citrate *versus* lactate — can then allow carbon flow within TCA to be probed. Gas-phase fragmentation (MS/MS) would also allow the position of the isotope to be established, although this is more readily achieved using NMR spectroscopy. Finally, labelling selectively in other places within glucose — [1,2-^13^C_2_]-d-glucose or [1,6-^13^C_2_]-d-glucose — are used for studying the pentose phosphate (PP) pathway. ^13^C flux analysis is also very important within cancer biology [57] as it allows understanding of cellular metabolism in terms of nutrient uptake (usually of glucose and glutamine), utilisation and secretion of lactic acid as this is related to the Warburg effect [58].

Nitrogen metabolism can be explored using similar approaches where natural ^14^N (99.6% abundance) is selectively substituted with the ^15^N stable isotope (0.4% abundance) within amino acids [59]. However, transamidase enzymes in many microbiological systems often remove the nitrogen group and the fate of the labelled ^15^N is harder to follow as it ends up in ammonium (NH_3_).

Microbial cell factories are excellent choices for synthetic biology as they are readily amenable to genetic engineering and some, such as *Pseudomonas putida*, have intrinsic metabolic flexibility to produce many fine chemicals like amino acid derived compounds, non-ribosomal peptides, polyketides, as well as rhamnolipids and terpenoids from renewable substates [60]. Armed with genome-scale metabolic reconstructions, mass isotopomers can be used to follow the flow of carbon or nitrogen to the desired product. This could be used for the identification of bottlenecks or alternative pathways that use that carbon or nitrogen. This has been nicely illustrated by Kukurugya et al. [61] who combined isotope tracers, metabolomics, fluxomics and proteomics to identify regulatory points (metabolic nodes) in *P. putida* that enable metabolic segregation of different carbon substrates. For those interested in learning more about multi-omics, which combines flux measurements within systems biology for biomanufacturing research please see a recent nice review by Chen et al. [62].

Finally, although not related to bioprocessing *per se*, Cortassa et al. [63] described similar approaches for translating metabolite profiles into sets of metabolic fluxes. They tested this with metabolite profiles obtained from *ex vivo* mice Langendorff-heart preparations perfused with glucose. They found that they could generate detailed kinetic fluxome models of glucose catabolic pathways including glycolysis, PPP, glycogenolysis and polyols. The authors suggest that this method can be used to diagnose, prevent, and treat metabolic diseases and they went on to use their fluxomics approach with fluxome calculations within central catabolism in diabetic hearts from mice to understand carbohydrate metabolism in diabetes further [64].

In summary, targeted metabolite analysis with quantification, metabolomics in its traditional sense and the adoption of stable isotope tracers (mass isotopomers) for flux analysis have improved synthetic biology and accelerated biomanufacturing to increased product yields. This combination of experimental data on small molecules with computational models of metabolism has improved engineering of host cell metabolism using tools such as genome shuffling and directed evolution. These developments have led to the increased production of high-value fine chemicals, reduced production of unwanted secondary by-products such as toxins and inhibitors, as well as reducing the energy costs in production [65].

## The application of metabolomics in human microbiome investigations

The fact that microorganisms are found everywhere and lay the foundation to sustain life on Earth, is undeniable. According to recent studies the number of microbial cells (referred to as microbiota or microflora) that inhabit our bodies are *at least equal*, if not more, than the number of human cells [66]. However, their collective genes (microbiome) at any one time is far greater than the entire human genome. The microbiota is generally described as the collection of microorganisms including archaea, bacteria, viruses, protozoa and fungi that inhabit the human body. There is mounting evidence from the literature demonstrating the crucial role of microbiota/microbiome towards human health and/or regulating the risk of various diseases. Given the complex relationship between microbial communities and the host, it is not surprising that disturbance of one often leads to disturbance of the other. For a recent in-depth review on the link between microbiome and human health, the reader is directed to an excellent review by Aggarwal et al. [67].

**Figure 4.**
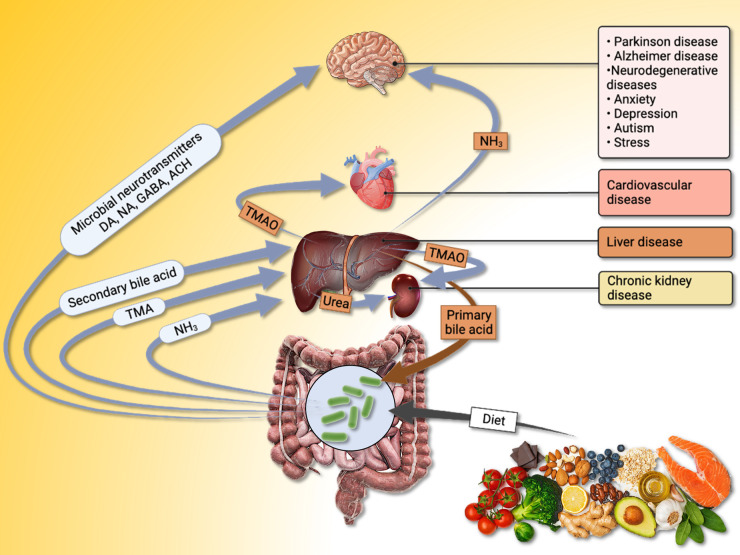
Diagram showing the potential interactions at the microbiota–gut–liver–brain axis. Ammonia (NH_3_) generated by the gut microbiota activity can be converted to urea in the liver and excreted by the kidneys through urine. However, dysfunction of liver, may result in higher levels of NH_3_ in systemic circulation, which may cross the blood–brain barrier causing hepatic encephalopathy. Choline is transformed to trimethylamine (TMA) by the gut microbiota, which is then delivered to the liver where it is converted into trimethylamine *N*-oxide (TMAO). Primary bile acids are produced in the liver, then transported to small intestine where they can be transformed into secondary bile acids by the metabolic activity of the gut microbiota, which may then be reabsorbed back into the blood circulation and transported to liver. The gut microbiota can contribute to the synthesis of neurotransmitters and/or their precursors, such as: dopamine (DA), noradrenaline (NA), gamma-aminobutyric acid (GABA) and adrenocorticotropic hormone (ACH).

Recent advancements in genetic sequencing technologies have provided an exponential amount of information regarding the genetic makeup and composition of different human microbiomes, which has transformed our understanding of the human microbiome composition. It is, however, worth noting that the language by which the microbiota interact with our body is generally through production of small biomolecules (metabolites). So, it is not surprising to observe an increase in the interest (Figure 4) and number of publications (Figure 1) on the application of metabolomics technologies and approaches to increase our understanding of such ‘chemical’ cross-talk at the functional level. Such approaches should assist our long-term goal of engineering these biological systems (microbiota) to perform desirable functions. In the following sections, we provide an overview of recent advancements and potential applications of metabolomic approaches to the area of microbiota–host interaction and its link with various human diseases.

### Metabolomics and microbiota-derived metabolites

A current hot topic is the intricate interaction between microbiota, especially those of the gut, and their host. This is primarily due to the progress in the fields of metabolomics and metagenomics, and the development of new technologies which has provided a much deeper understanding of this interaction and its crucial role towards human health and the development of various diseases such as, obesity, type 2 diabetes mellitus, Alzheimer's, cardiovascular and non-alcoholic fatty liver, to name a few [68]. The majority of these interactions occur through the release of a variety of metabolites with a wide range of bioactivities. Thus, comprehensive detection and identification of these metabolites is pivotal to advancing our understanding of their potential function and effects on the host.

In the past two decades, the application of MS-based metabolomics technologies has played an instrumental part in achieving this goal. For instance, in a recent study by Dekkers et al. [69], a combination of LC–MS-based metabolomics and metagenomics sequencing techniques were employed to investigate the link between metabolites in plasma (plasma metabolome) and the gut microbiota in 8583 participants, they reported that over 58% of the variance in the plasma metabolome of the participants could be explained based on the gut microbiota profiles. This study also reported strong associations between multiple metabolites and certain species of bacteria, such as the level of several *p*-cresol metabolites (*p*-cresol glucuronide and *p*-cresol sulfate), a well-known uraemic toxin and a microbially derived metabolite produced from tyrosine metabolism in the gut [70], being linked to various strains of *Faecalibacterium prausnitzii*. Talmor-Barkan et al. [71] also employed a multi-omics approach to investigate the link between the serum metabolome and gut microbiome in patients with acute coronary syndrome (ACS). This study reported the depletion of a previously unknown species of bacteria (*Clostridiaceae* family) in the ACS patients, compared with the healthy controls, this was found to be highly associated with changes in the level of multiple circulating metabolites including *p*-cresol sulfate and *p*-cresol glucuronide, several of which were previously reported to be linked with coronary artery diseases (CAD). Finally, in a large study leveraging 9000 individuals Wilmanski et al. [72] demonstrated that starting in mid-to-late adulthood the gut microflora and hence microbiomes become increasingly unique to individuals with age. This coupled with the production of indole derivatives from microbial tryptophan metabolism may affect host physiology.

Based on the findings of similar studies reported in the literature, the microbiota-derived metabolites (microbial exposome) can be grouped into three main categories: (i) metabolites that are produced by the gut microbiota through *de novo* synthesis (e.g. polysaccharide A, ATP), (ii) metabolites that are produced via interaction between the microbiota and the dietary and/or pharmaceutical components (e.g. short-chain fatty acids (SCFAs), NH_3_, trimethylamine *N*-oxide (TMAO) and indole derivatives), and (iii) host-produced metabolites that are modified through interaction with the gut microbiota (e.g. taurine, secondary bile acids) [71,73]. Below, we have provided examples of microbiota-derived metabolites and their link to various human diseases.

#### Trimethylamine *N*-oxide

Recent studies have reported major alterations in the gut microbiota community and their associated metabolites in patients with CAD [74], for example TMAO. Trimethylamine (TMA) is a product of l-carnitine and choline (dietary source) metabolism by the gut microbiota. The synthesised TMA can then be transported to the liver where it is converted to TMAO and enters the blood circulation (Figure 4). Numerous studies have demonstrated the link between increased TMAO and a wide range of human diseases such as obesity [75], diabetes [76], cardiovascular disease [77] and kidney failure [78]. A notable example is the large-scale (*n = *648) study by Mano et al. [79], which used a cross-sectional multi-omics (metagenomics, MS-based metabolomics and proteomics) approach to investigate the link between the microbiome patterns and TMAO levels. The authors identified several microbiota-derived metabolites associated with TMAO and demonstrated the influence of diet on the gut microbiota composition and TMAO levels. Increased TMAO levels have also been strongly associated with renal dysfunction [80]. Compromising the TMAO renal clearance may result in higher circulating TMAO levels causing chronic kidney disease and coronary atherosclerosis [81]. However, recent studies have reported promising results using berberine (BBR), an isoquinoline alkaloid plant extract used in traditional Chinese medicine, to reduce TMA/TMAO production [82]. The authors investigated the effects of BBR in mice models with atherosclerosis and applied a targeted LC–MS/MS method to monitor and quantify the circulating TMA and TMAO levels in serum. The BBR treatment in this study resulted in the remodelling of the gut microbiota, reduced atherosclerosis and lowered choline to TMA conversion and consequently lowering TMAO levels.

#### Bile acids

Another class of metabolites that has attracted significant attention and established links to human–microbiota interaction are bile acid metabolites. Primary bile acids (BAs) (e.g. cholic acid and chenodeoxycholic acid) are originally synthesised by hepatocytes in the liver and deposited in the small intestine, where they can be reabsorbed and transported back into the liver (enterohepatic circulation), or alternatively transformed into secondary BAs (e.g. ursodeoxycholic acid and deoxycholic acid) by the metabolic activity of the gut microbiota. BAs have been shown to be linked to various signalling molecules such as vitamin D receptors and farnesoid X receptors, which can affect the hosts metabolism by modulating glucose, lipid and energy balance. According to the study by Swann et al. [83] which applied a targeted UPLC-MS approach for the detection and comparison of BAs levels in germ-free and antibiotics-treated rats compared with conventional rats, the detected BAs diversity was much lower in germ-free and antibiotic-treated rats. In addition, the BA signatures detected in the kidney, heart, plasma and liver was clearly affected by the presence/absence of the gut microbiota. These findings suggest a potentially broader signalling role of BAs, while highlighting the influence of the microbiota activity on the homeostasis of its host. Additionally, primary bile acids have traditionally been known to conjugate with two amino acids (glycine and taurine) in the liver. Recent research has identified that other amino acids also conjugate with primary bile acids which leads to potentially more signalling roles of BAs [84].

#### Ammonia

The gut microbiota contributes to ammonia production via metabolism (e.g. deamination) of proteins, peptides and amino acids present in the diet. The regulation of ammonia levels in the human body is via the urea cycle in the liver, where ammonia is converted to urea and transported to the kidneys for excretion via urine (Figure 4). Thus, alteration in liver function (e.g. cirrhosis) can disrupt this balance and result in higher levels of ammonia in systemic circulation (hyperammonemia), which may cross the blood–brain barrier leading to a condition known as hepatic encephalopathy (HE). Multiple recent studies have reported alterations in the gut microbiota composition and function in cirrhosis and HE patients [85]. However, a recent study reported attenuated hyperammonemia in HE animal models upon faecal microbiota transplantation [86]. Iebba et al. [88] employed a combination of 16S rRNA sequencing and NMR-based metabolomics analysis of faeces, caecal biopsies and peripheral/portal liver blood samples from cirrhosis patients to identify significant metabolites and bacterial species associated with this condition. Several bacterial species, mainly *Pseudomonas* and other *Proteobacteria* members, were identified as the main translocators in the portal blood. Moreover, a reduced level of bile acids was detected in cirrhosis patients which correlated with a reduction in *Clostridium* species, while higher levels of *Prevotella copri* was positively correlated to higher TNFα levels and worsening of the systemic inflammation. The authors also reported a positive correlation between patients with HE and increased level of *Methylobacterium extorquens*, an opportunistic pathogen capable of oxidising methanol to formaldehyde. These findings were confirmed further by the detection of higher faecal-methanol levels in HE patients, which may have supported the proliferation and increased activity of *M. extorquens* in the peripheral blood and its contribution to systemic disease. TMA was also found to be higher in faecal samples of these patients, which as described in the sections above, could lead to higher TMAO levels in liver and increasing the risk of cardiovascular diseases.

#### Microbial-derived neurotransmitters

Several recent studies have reported an imbalance of the gut microbiota in patients with neurological disorders, such as: Parkinson's disease, Alzheimer's disease, anxiety and autism [18,89]. Increasing evidence from the literature indicates the significance and potential roles of microbiota-derived metabolites (e.g. SCFAs, neurotransmitters and their precursors) in regulating brain function and cognition. This bidirectional interaction is generally known as the gut–brain axis. Several studies, using germ-free mice models, have reported significant changes in the serum and faecal levels of neurotransmitters including, acetylcholine, serotonin and gamma-aminobutyric acid (GABA), and their precursors (e.g. choline and tryptophan), in the absence of gut microbiota [90–92]. It is also worth noting that although the blood–brain barrier does not usually allow for large molecules, such as neurotransmitters, to penetrate the brain small precursor molecules such as amino acids are easily transported [94]. A recent study by Xiao and colleagues [94], investigated the effects of faecal transplantation from human donors with autism spectrum disorder (ASD), and healthy individuals (control group), into germ-free mice models. Samples were taken after three weeks for sequencing of the caecal content, and untargeted metabolomics analysis of the serum, caecum and prefrontal cortex. The study reported changes in the detected levels of serotonin and tryptophan between the ASD and control donors, while demonstrating that the faecal microbiota transplantation of the ASD donors also resulted in alteration of serotonin and tryptophan metabolism, changed the microbiota community and developed ASD-like behaviour in the germ-free mice models. Using a similar approach Zhu et al. [95], investigated the effects of faecal microbiota transplantation from patients with schizophrenia and healthy donors into germ-free mice. The authors reported behavioural abnormalities such as impaired learning, and psychomotor hyperactivity in mice models that received transplantation from the schizophrenic patients. In addition, these mice models showed an elevated kynurenine–kynurenic acid pathway of tryptophan degradation, as well as increased extracellular dopamine and 5-hydroxytryptamine in the prefrontal cortex and hippocampus, respectively.

### Future perspectives

Metabolomics and multi-omic approaches have advanced our understanding of microbiota–host interaction, evidenced by the number of publications and example articles discussed above. The validation of methods for sample collection and metabolite extraction are important considerations and this is especially vital when it comes to analysing faecal samples and the gut metabolome. Most importantly, employment of multiple complementary metabolomic technologies (e.g. GC–MS, LC–MS and NMR spectroscopy) and its integration with metagenomics strategies is crucial in providing a complete microbiome coverage and establishing the function of potential key players in the gut microbiota.

A major challenge in microbiome–metabolomics research is the ability to differentiate the source of the detected metabolites, in other words to determine whether they are derived from the host, microbial activity or the diet with many studies reporting microbially derived metabolites in human blood [96], phytochemicals from food [97], as well as drugs and their metabolites [98]. Despite the use of germ-free mice models and antibiotics-treated models by numerous studies to identify microbiota-derived metabolites, so far, our understanding of the source and functional properties of these biomolecules remains limited. Hence, future efforts should focus on the development of comprehensive microbiota-derived metabolite databases, such as Exposome-Explorer [73], to guide metabolite identification, while also attempting to incorporate bioinformatics pipelines and statistical tools with existing biological knowledge and metabolic potential of the known microbiota, such as MetOrigin [99]. We envisage the development of these tools will enable the systematic investigation of microbiota–metabolite interactions, and detangling of host–gut-microbial processes within complex mammalian-microbe hydrid superorganism systems [100,101] which play pivotal roles within health and disease [102].

## Concluding remarks

While one can debate that analytical biochemistry has always been a flavour of metabolomics, the discipline of metabolomics was formally established in the later part of the 20th century [103] and clearly has its roots in the microbial and plant sciences [104] as well as mammalian systems [8,105]. Experiments by Horning, Horning and colleagues predate these investigations (and indeed the birthdays of all of these authors on this review!) where gas chromatography was used in the 1960s to separate a wide range of metabolites found in urine or tissue extracts [106], and these authors even referred to these measurements as ‘metabolic profiles’ [88] 27 years before the word metabolome first appeared in the literature [103].

The field of metabolomics has matured over the past 25 years resulting in the development of robust and reproducible workflows that have driven the application of metabolomics to new areas across the whole of the biological sciences. Despite significant progress in standardisation across the whole of the metabolomics and lipidomics workflows being achieved by international consortia [32,33,107], there still remain significant challenges in correct annotation of metabolites [108] and ensuring that metabolite identifications are fact based [109]. In addition, for many systems biology and synthetic biology models there is a need for accurate quantification of metabolites with metabolomes, and this is far from straightforward [85,110].

In this review we have discussed the application of metabolomics in two complementary areas that have benefited from these developments. Synthetic biology where the application of metabolomics in the DBTL cycle has resulted in increased production of high-value fine chemical and the concomitant reduction in secondary by-products. The second area addresses human-microbe research which has grown in interest due to the role of the microbiome in health and disease. The potential for metabolomics is recognised but the development of specific databases and bioinformatics tools are required to detangle the host–gut-microbial processes. We expect many future discoveries to result from future research in these two and other areas of research across the biological sciences.

## Open Access

Open access for this article was enabled by the participation of University of Liverpool in an all-inclusive *Read & Publish* agreement with Portland Press and the Biochemical Society under a transformative agreement with JISC.

## Abbreviations

ACS: acute coronary syndrome
ASD: autism spectrum disorder
BAs: bile acids
BBR: berberine
CAD: coronary artery diseases
CHO: Chinese hamster ovary
DBTL: Design–Build–Test–Learn
EI: electron ionisation
GC–MS: gas chromatography–mass spectrometry
HE: hepatic encephalopathy
LC–MS: liquid chromatography–mass spectrometry
MSI: metabolomics standards initiative
NH_3_: ammonia
NMR: nuclear magnetic resonance
PP: pentose phosphate
QA: quality assurance
QC: quality control
RT: retention time
SCFAs: short-chain fatty acids
TMA: trimethylamine
TMAO: trimethylamine *N*-oxide
